# Identifying the factors affecting bike-sharing usage and degree of satisfaction in Ningbo, China

**DOI:** 10.1371/journal.pone.0185100

**Published:** 2017-09-21

**Authors:** Yanyong Guo, Jibiao Zhou, Yao Wu, Zhibin Li

**Affiliations:** 1 Jiangsu Key Laboratory of Urban ITS, Southeast University, Nanjing, China; 2 Department of Civil Engineering, University of British Columbia, Vancouver, British Columbia, Canada; 3 School of Civil and Transportation Engineering, Ningbo University of Technology, Ningbo, China; Shanghai University of Finance and Economics, CHINA

## Abstract

The boom in bike-sharing is receiving growing attention as societies become more aware of the importance of active non-motorized traffic modes. However, the low usage of this transport mode in China raises concerns. The primary objective of this study is to explore factors affecting bike-sharing usage and satisfaction degree of bike-sharing among the bike-sharing user population in China. Data were collected by a questionnaire survey in Ningbo. A bivariate ordered probit (BOP) model was developed to examine simultaneously those factors associated with both bike-sharing usage and satisfaction degree of bike-sharing among users. Marginal effects for contributory factors were calculated to quantify their impacts on the outcomes. The results showed that the BOP model can account for commonly shared unobserved characteristics within usage and satisfaction of bike-sharing. The BOP model results showed that the usage of bike-sharing was affected by gender, household bicycle/e-bike ownership, trip model, travel time, bike-sharing stations location, and users’ perception of bike-sharing. The satisfaction degree of bike-sharing was affected by household income, bike-sharing stations location, and users’ perception of bike-sharing. It is also found that bike-sharing usage and satisfaction degree are strongly correlated and positive in direction. The results can enhance our comprehension of the factors that affect usage and satisfaction degree of bike-sharing. Based on the results, some suggestions regarding planning, engineering, and public advocacy were discussed to increase the usage of bike-sharing in Ningbo, China.

## Introduction

China was famous as the Kingdom of bicycles in the 1970s due to the heavy reliance on cycling for mobility, however, with rapid economic growth and motorization, bicycle use has significantly decreased. In recent years, owing to traffic congestion, air pollution and safety problems caused by motorized vehicles in most Chinese cities, the potential benefits of bicycle use for short distance trips was encouraged [1–3]. Bicycles occupy less road space and produce fewer emissions as compared to motorized modes, and thus their use in urban areas is generally recognized as an environmentally friendly mode of transport [4,5].

As an emerging transport mode, bike-sharing—the shared use of a bicycle fleet—increases bicycle use. It provides a variety of pick-up and drop-off locations, free of use (usually within one hour), and self-service, making it more convenient and attractive to users. In addition, serving as a complement to other urban transit systems, bike-sharing offers an efficient solution to the “last mile” problem [6]. They can connect from/to transit stations to/from their final destination/home, reducing pressure on expanding transit services [7]. Trips with bike-sharing can be single or round-trip, allowing the bicycles to be used for one-way transport and for multi-modal connectivity. Bike-sharing is viewed as an economic, efficient, and healthy transport mode in a dense urban environment.

**Fig 1:**
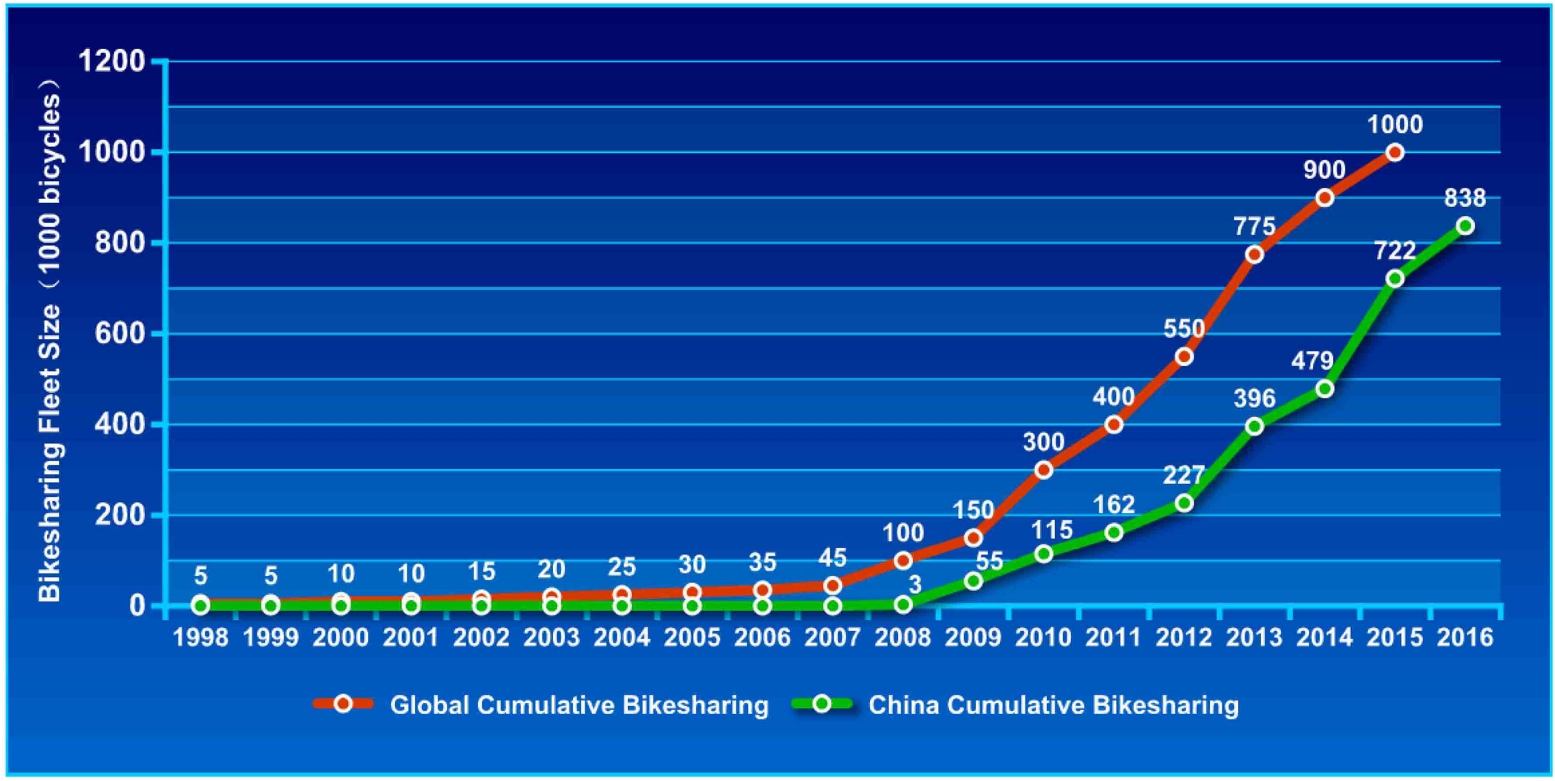
The rapid growth of bike-sharing programmes.

Due to the advantages and benefits of bike-sharing, it has spread globally [8–10]. Paris launched Europe’s largest bike-sharing programme with over 20,000 bicycles in 2007. In 2013, New York launched North America’s largest scheme with 10,000 bicycles [11]. China has the world’s largest bike-sharing schemes, and leads the greatest growth of bike-sharing around the word [11,12]. Fig 1 shows the rapid growth of these bike-sharing programmes. There are currently approximately 750,000 shared bicycles in China and the number is estimated to increase to nearly a million [11,13]. The expansion of bike-sharing in China is supported by Transit Priority Policies as proposed by the Ministry of Housing and Urban and Rural Development (MHURD) in 2004, which aimed at reducing carbon dioxide emissions [14]. In the implementation of this strategy, the bike-sharing system has spread across the country in recent years, from the capital Beijing to a small countryside town (Yonglin) in Zhangjiagang [15].

**Fig 2:**
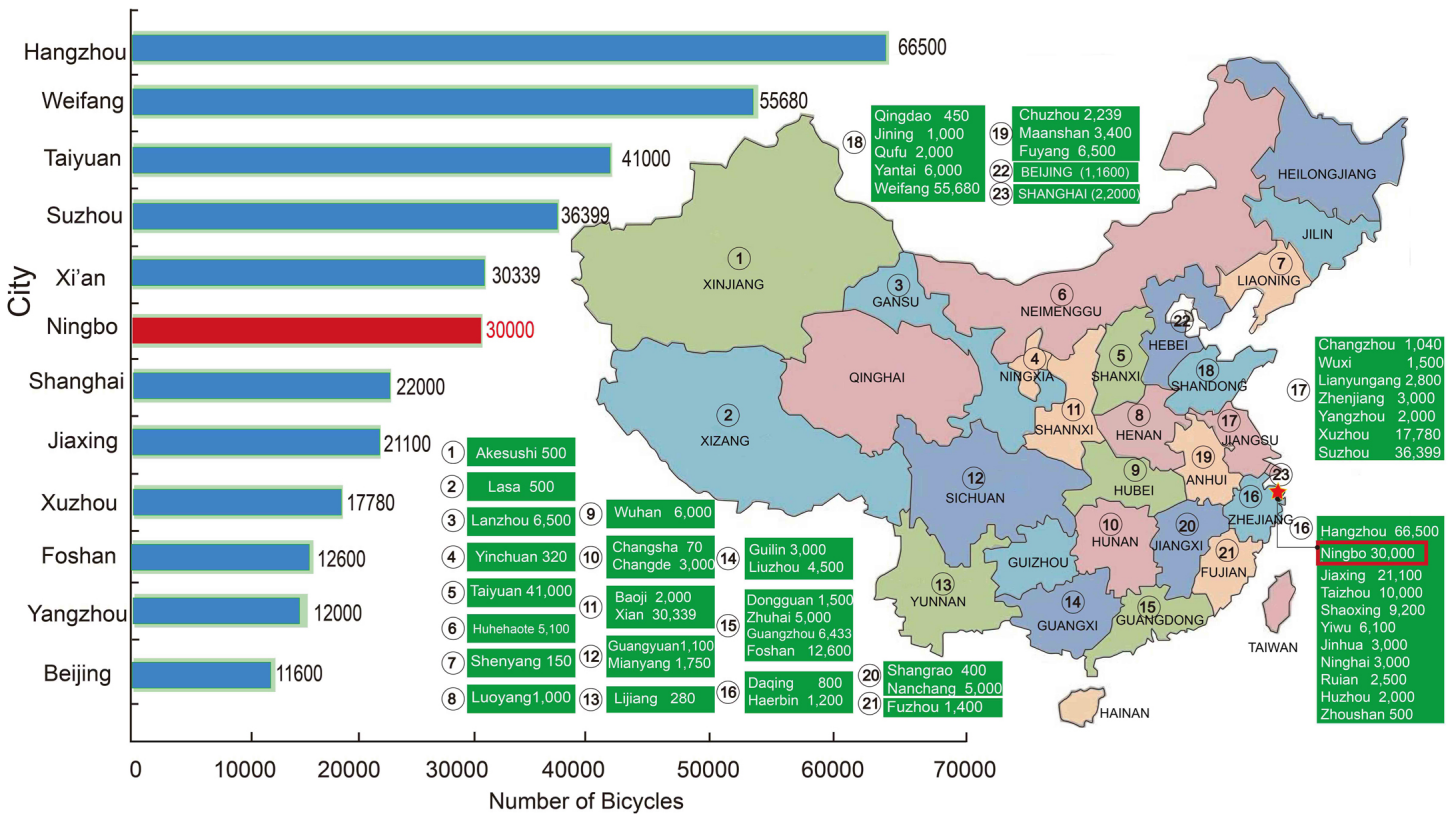
The boom in bike-sharing programmes in China (by the end of 2015).

Early in 2005, China launched the first bike-sharing system in Beijing, which is not information technology (IT) based [14]. Owing to the transportation reforms enacted for the 2008 Olympics, fleet sized growth varied from 5,000 to 10,000. However, the system failed by 2010 as a result of bad user experiences, lack of stations, and poor maintenance of the bicycles [16,17]. Since then, the IT-based bike-sharing systems have boomed in other Chinese cities. Hangzhou opened the first IT-based public bike-sharing programme in 2008 with 2,000 bike-sharing stations and 50,000 bicycles [14]. As of January 2015, there are approximately 140 cities which have launched a bike-sharing programme in China (Fig 2) [18].

Although bike-sharing flourishes across cities in China, the low usage of this mode raises concerns. Shenyang, for example, has even given up its bike-sharing programme after three years of operation because of low usage [19]. As such, a crucial issue is the recognition of factors affecting bike-sharing usage. A better understanding of the factors influencing bike-sharing usage and the bike-sharing user satisfaction degree can help to develop improvements for promoting its usage and improving the performance of existing bike-sharing systems.

The primary objective of this study is to understand factors affecting bike-sharing usage and user satisfaction degree of the bike-sharing. More specifically, we evaluate how the demographic characteristics, travel patterns, built environments, and user perceptions affect the usage and satisfaction degree of bike-sharing. This paper applies a bivariate ordered probit (BOP) modelling approach to explore and examine the potential factors affecting both bike-sharing usage and satisfaction degree of bike-sharing simultaneously. The analysis is demonstrated using a case study from Ningbo, China.

## Literature review

### Bike-sharing programmes

A review of the literature shows that bike-sharing programmes can be categorized into four generations based on their operational and logistical development [14]. The first generation of bike-sharing programmes can be found in Europe and dates back to the late 1960s, with the famous “White Bicycle System” in Amsterdam, Netherlands. The second generation known as the “Coin Deposit Systems” required users to insert a refundable deposit to unlock and use a bicycle. Unfortunately, the two programmes failed due to the number of stolen and vandalized bicycles, and lack of time constraints on their use.

It became widely recognized, from the third generation “IT-based systems”, that designated docking stations and smart technology for bicycle check-in and check-out, such as the large-scale bike-sharing system—Velo’v—launched in Lyon, France in 2005 [20]. Since then, the number of bike-sharing programmes has grown exponentially around the world with the concerns about global motorization, traffic congestion, and greenhouse gas emissions. The fourth generation, known as “Demand Responsive, Multimodal Systems”, integrated with larger public transport systems via smart cards as a key feature. The new generation may also introduce kinds of bicycles such as electric bicycles and bicycle redistribution systems.

Lately, more studies on bike-sharing have been reported with bike-sharing’s widespread expansion [8, 21, 22]. A study by Shaheen *et al*. [6] analysed the history and evolution of bike-sharing in Europe, the Americas, and Asia. They discussed bike-sharing business models and lessons learned, highlighting the social and environmental benefits of bike-sharing. In subsequent studies, Shaheen *et al*. [8] and Parkes *et al*. [9] investigated bike-sharing usage in North America. They documented the state of IT-based public bike-sharing programmes in the USA and Canada and highlighted emerging trends for prospective start-ups. The studies revealed that the most common bike-sharing trip purpose is work- or school-related, indicating that bike-sharing was used for commuting purposes.

A recent study by O’Brien *et al*. [23] analysed bike-sharing characteristic data from 38 systems located in Europe, the Middle East, Asia, Australasia, and the Americas from a global perspective. By analysing the variation of occupancy rates over time, and comparing across the system’s extents, O’Brien *et al* [23] presented a classification of bike-sharing systems, based on their geographical footprint and diurnal, day-of-week, and spatial variations in occupancy rates. Fishman *et al*. [10] analysed the effects of bike-sharing on car use. Through an examination of large-scale surveys and trip data from bike-sharing programmes in Melbourne, Brisbane, Washington, D.C., London, and Minneapolis/St Paul, Fishman *et al*. [10] examined the degree to which car trips were replaced by bike-sharing. There was evidence from these studies that the benefits of bike-sharing can also be expanded to improve travel connectivity, and reduce driving and emissions. Recent bike-sharing research concerning bike-sharing at an urban level is also available [21,24].

### Contributory factors related to bike-sharing usage

Previous studies captured the determinants of bike-sharing usage by using actual bicycle usage data. The studies examined the effects of transportation infrastructure, bike-sharing facilities, land-use and built environment characteristics, and temporal characteristics on bike-sharing usage. For example, a recent study by Campbell *et al*. [12] concluded that the choice to bike-share was most sensitive to measures of effort and comfort. It was found that bike-sharing demand was negatively affected by trip distance, temperature, precipitation, and poor air quality. Fishman *et al*. [25] reported that the lack of accessibility/spontaneity, overnight closure of the system, and an inability to sign-up easily with a credit card swipe, were significant barriers to bike-sharing usage. Zhao *et al*. [15] and Rixey [26] demonstrated that bike-sharing ridership increased with increasing numbers of bike-sharing facilities such as the number of docking stations and public bikes. Bachand-Marleau *et al*. [27] reported that bike-sharing system usage increased when there were more bicycle facilities (such as bicycle lanes, bicycle paths) near a bike-sharing station.

Several studies indicated that the land-use and built environments, such as the presence of metro and bus stations, restaurants, and universities, contributed to bike-sharing usage [27]. For instance, Wang *et al*. [28], Faghih-Imani *et al*. [29], and Hampshire *et al*. [30] identified that the number of restaurants in the vicinity of a bike-sharing station increased its usage. Rixey [26] and Wang *et al*. [31] corroborated their findings that the presence of a paved trail or bikeways in the vicinity of the station would increase bike-sharing usage. Bachand-Marleau *et al*. [27] and Faghih-Imani *et al*. [29] found that the usage would decrease when a bike-sharing station is located farther from the central business district (CBD). Studies examined the impact of temporal characteristics found that people tended to use public bicycles more on weekdays than weekends, and the bike-sharing system was more predominantly used during evening peak hours relative to other times of the day [29].

The literature review indicated that previous studies focused on bike-sharing usage were undertaken at an aggregate level. Most bike-sharing studies rely on ridership data but do not provide much information about bike-sharing users. In addition, little is known about the underlying factors that affect the satisfaction degree of bike-sharing. Furthermore, there is a paucity of research discerning the interrelationships between the bike-sharing usage and satisfaction of bike-sharing, and the contributory factors at a disaggregated level using robust statistical techniques. This study developed a BOP model to examine the potential factors affecting both bike-sharing usage and satisfaction degree of bike-sharing simultaneously. Potential correlates between bike-sharing usage and user satisfaction degree of bike-sharing were also examined.

## Overview of bike-sharing programme in Ningbo

Ningbo is one of the biggest cities on the east coast of China with a population of 5.8 million and an area of 9,817 km^2^ by the end of 2014. The city is also one of the richest cities in China. In 2014, the gross domestic product (GDP) reached 760.25 billion RMB ($122.55 billion), a 7.6% increase from the previous year [32].

**Fig 3:**
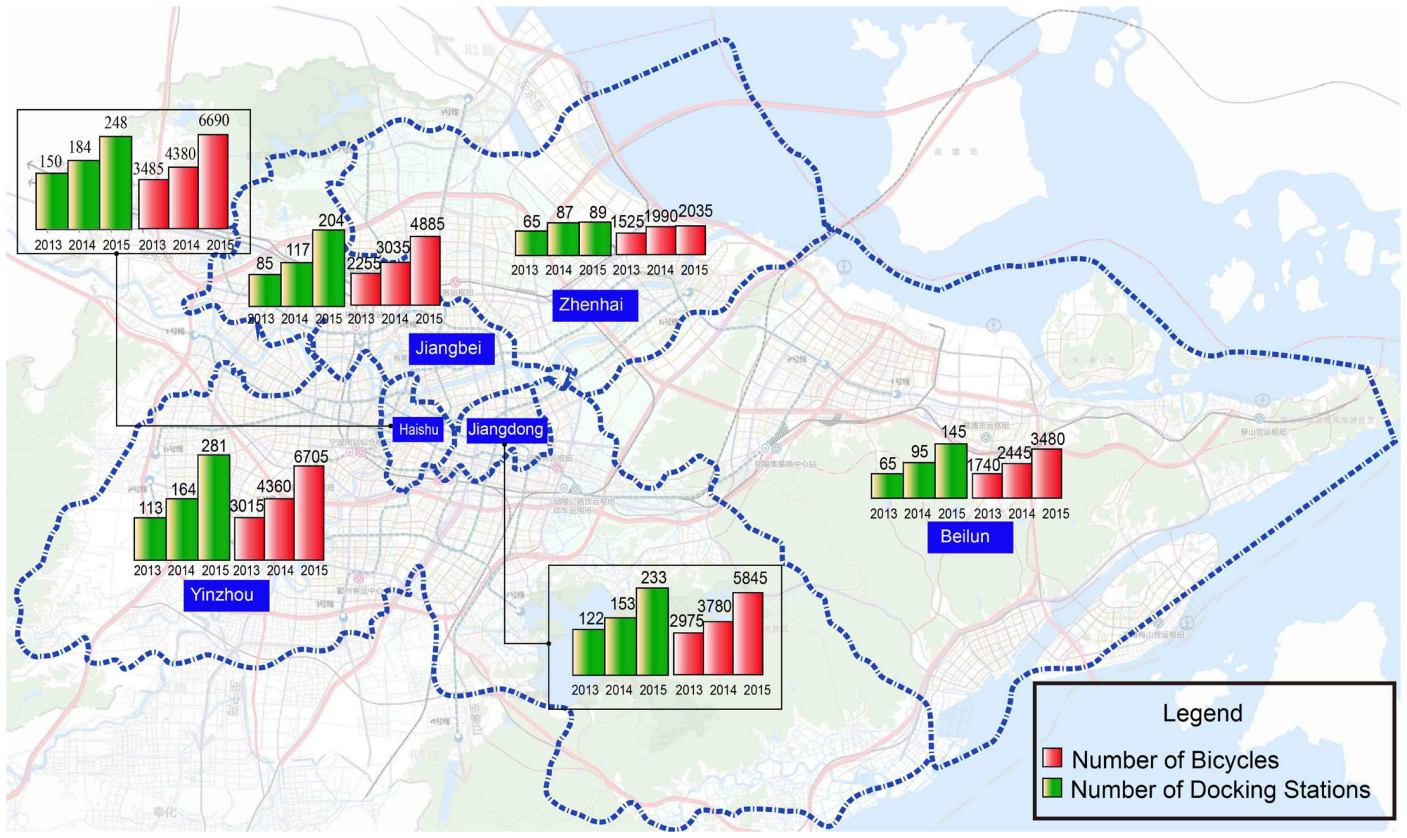
Bike-sharing system (bicycles and docking stations) deployment in Ningbo.

The public bike-sharing system in Ningbo was launched on World Car Free Day (22 September) in 2013 by Local Governments and it is operated by a government-owned company—the Ningbo Public Transport Corporation. The local Ningbo government invested 96 million RMB ($15.5 million) to support the programme [33]. The construction of the bike-sharing programme is divided into three phases. Phase one in 2013 consists of 600 docking stations and 15,000 bicycles deployed in the downtown area, phase two from 2014 to 2015 consists of 200 docking stations and 5,000 bicycles expanded to six core districts, and phase three from 2016 to 2017 consists of 400 docking stations and 10,000 bicycles. By the end of 2017, there will be approximately 1,300 docking stations and 30,000 bicycles covering the entire urban area. The bike-sharing system deployment is shown in Fig 3.

The Ningbo bike-sharing system adopts third generation bike-sharing techniques. It uses designated docking stations and a smart card for automated check-in and check-out. With the aim of enhancing the link between public bicycle and transit, the bike-sharing programme allows users to rent a bicycle using their public transit card for a 10% discount. The current smart card requires a 200 RMB ($32 USD) deposit for bike-sharing use. The first hour of use is free, and this hour is followed by incremental pricing in which users pay an additional 1 RMB ($0.16) for the second hour, 2 RMB ($0.32) for the third hour, and 3 RMB ($0.48) for each hour thereafter [32]. Due to its low cost, the service enabled 90% of total trips to be free of charge. In addition, Ningbo bike-sharing provides a 24-h service station and supports one-way trips and intermodal transfers.

**Fig 4:**
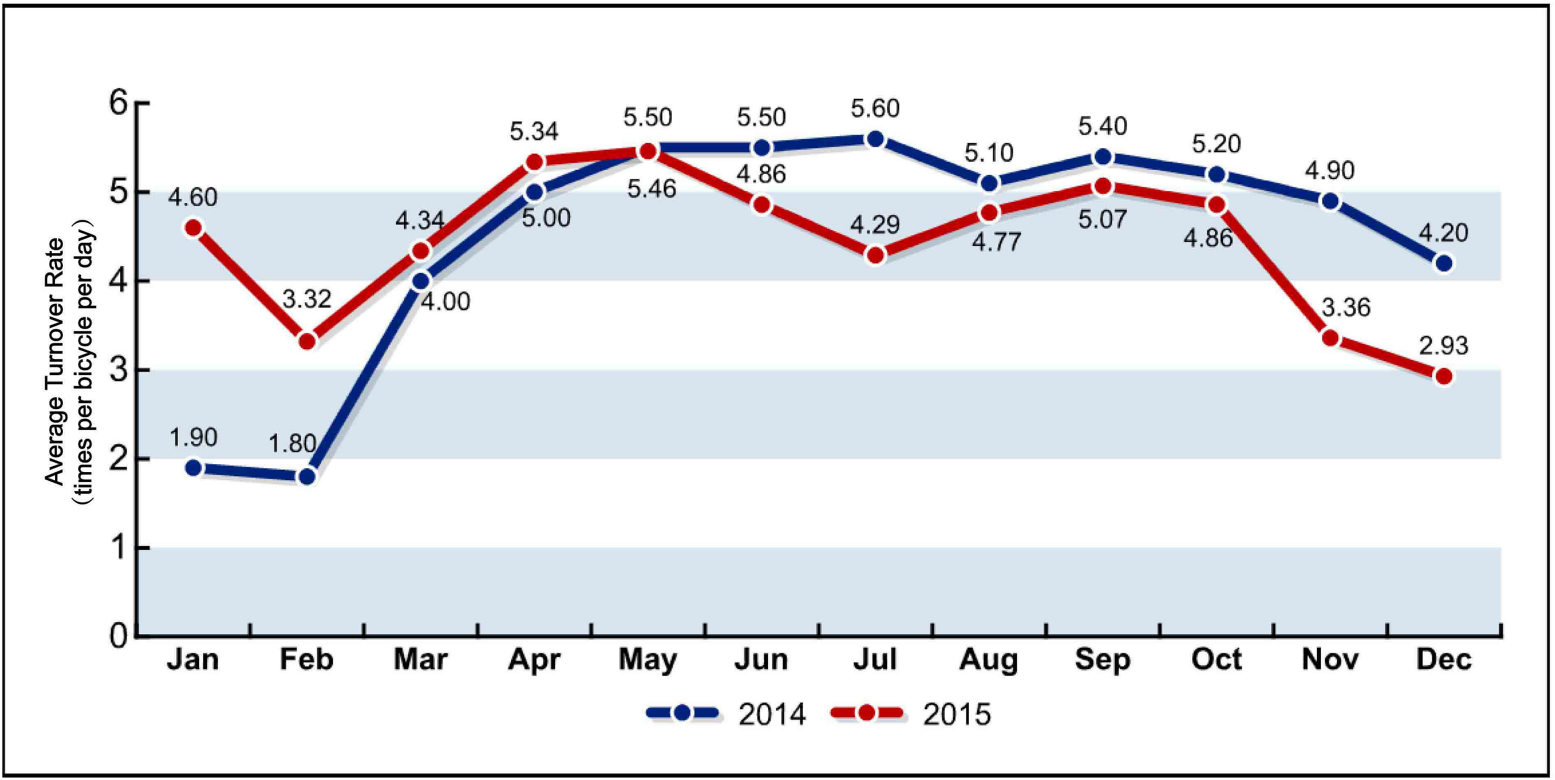
Average daily turnover rate of bike-sharing across all months.

**Fig 5:**
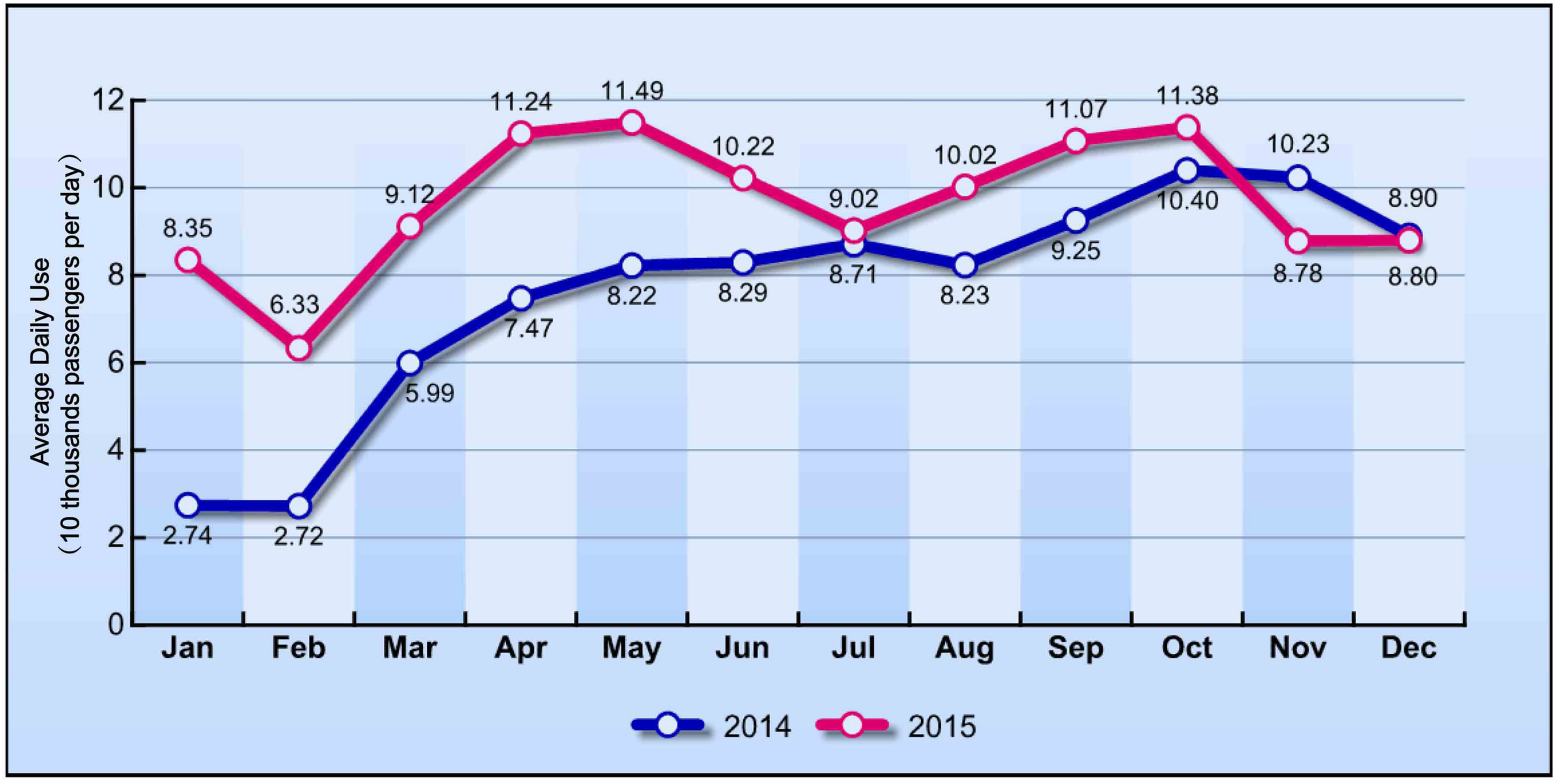
Average daily use of bike-sharing across all months.

As of October 2016, the Ningbo bike-sharing system operated 1,215 docking stations and 29,635 bicycles [34]. The bike-sharing members registered via IC smart card numbered over 250,000. In the last two years, the average turnover rate varied from 1.8 to 5.6 with a mean of 4.47 times per bicycle per day. The daily use varied from 27,200 to 114,900 with a mean of 86,800 passengers per day. The average turnover rate and daily use of the Ningbo bike-sharing system were comparable with the average value across China reported in [15], but much lower than that of the successful bike-sharing programmes in Shanghai, Guangzhou, and Shenzhen. Figs 4 and 5 show the average daily turnover rate and daily use across each month in the last two years. The service radius of the bike-sharing station is between 150 m and 200 m. Bicycles were deployed at a density of 80 to 100 per station near the transit station, 50 to 100 per station near the large bus loop, and 20 to 40 per station at other places such shopping centres, other neighbourhoods, and bus stations [35]. The station billboards and bicycle advertisements are the main revenue source for the scheme.

## Data and methods

### Conceptual model

**Fig 6:**
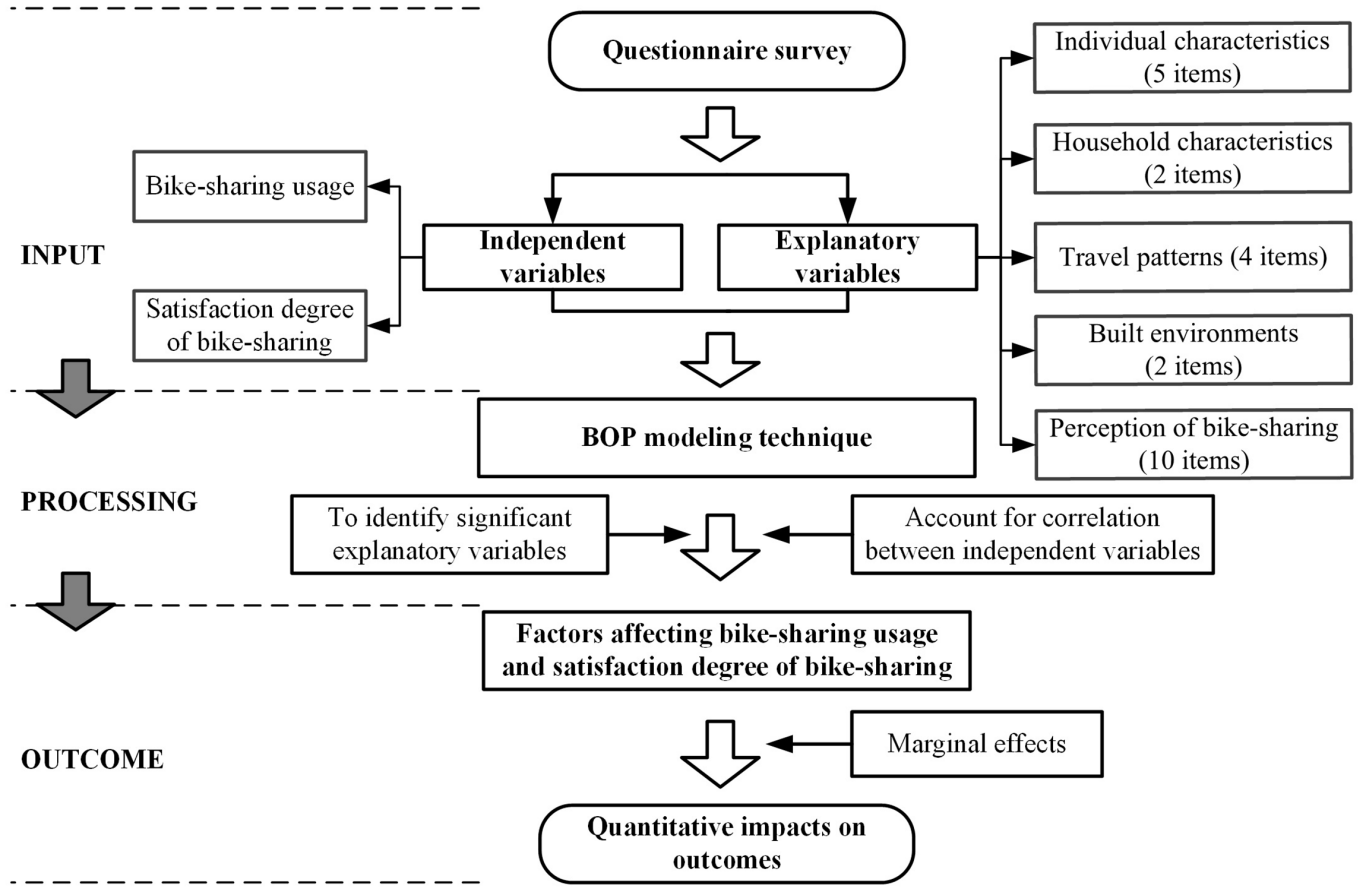
The conceptual model procedure.

A conceptual model, including three procedures—input, processing, and outcome, is proposed in order to explore the factors affecting bike-sharing usage and degree of satisfaction as shown in Fig 6. The input of the model are independent and explanatory variables. A questionnaire survey was used to collect bike-sharing usage and satisfaction data. As well, the explanatory variables that includes individual characteristics, household characteristics, travel patterns, built environments, and perception of bike-sharing are collected by the questionnaire survey. In the processing procedure, the bivariate ordered probit (BOP) modeling technique was used to identify the significant factors affecting bike-sharing usage and degree of satisfaction. This modeling technique can account for the correlation between independent variables. The outcome of the conceptual model is the factors related to bike-sharing usage and degree of satisfaction. Subsequently, marginal effects of the interested factors (significant variables) were calculated to quality their impacts on the usage and satisfaction levels.

### Data collection

Questionnaire surveys, which have been widely used in transportation engineering research, were designed to collect bike-sharing usage and satisfaction degree information in Ningbo. The questionnaire survey was carried out among bike-sharing memberships based on their bike-sharing experiences. The survey was a part assignment within *IMPROVEMENT PROGRAMMES FOR NINGBO BIKE-SHARING USAGE* launched by the Ningbo Transportation committee (NBTC) in 2015. It was conducted in the six core districts of Ningbo: Haishu, Jiangdong, Jiangbei, Zhenhai, Yinzhou, and Beilun. This study was approved by the Academic Committee of Ningbo University of Technology.

The questionnaire was designed based on an extensive review of the literature and the results of pre-conducted focus group discussions. Before the formal survey was launched, a pre-test of 30 bike-sharing memberships was administered to identify potential problems with the questionnaires and to prevent biases. According to the feed-back, the initial questionnaire was revised to make the questions clearer. Although the self-reported survey has its limitations, particularly with regard to some of its subjective descriptions, surveys offer the opportunity to supplement analysis with detailed demographic data. The approach provides us with previously omitted personal characteristics such as age, gender, household income, and perception of bike-sharing, and their impacts on usage and satisfaction of bike-sharing. Furthermore, cross-check questions were set in order to filter out any self-reporting bias. The questionnaire included five parts as presented by the following abbreviated description.

In the survey, respondents were asked about their (a) bike-sharing usage component on a three-point Likert scale from one (occasionally) to three (frequently); (b) the level of satisfaction degrees of bike-sharing with three-point Likert-type answers from one (bad) to three (good); (c) travel patterns including trip mode, travel time, trip purpose at weekdays and weekends, and built environment such as bike-sharing station and bus stops available within 500 m from home or workplace; (d) perception of bike-sharing such as familiarity with bike-sharing, satisfaction with bike-sharing fees, saving travel costs by bike-sharing, and easy to check-in and check-out; and (e) demographic information such as gender, age, education level, occupation, monthly income, and car/bicycle/e-bike numbers in household.

Students from the School of Transportation, Ningbo University of Technology (NBUT) were hired to conduct the face-to-face surveys in May and June, 2015, and the authors were not involved in collecting survey data. The respondents were asked by investigators if they consented to participate in the anonymous study, and the participants gave their verbal consent to the survey. The surveys were administered on both workdays and weekends to collect a broad range of respondent types. During the survey, questionnaire investigators were placed at the bike-sharing docking stations near bus/metro stations, shopping centres, and on busy street corners. The random sampling technique was employed in selecting the bike-sharing users. The investigators were instructed to randomly select every fifth person (those hold a bike-sharing smart card and older than 12 years) who passed through their sampling domain. Investigators remained nearby to explain any questions to the respondents during the survey. After completing the questionnaire, each respondent was offered a small gift as a token of appreciation. A total of 1,200 questionnaires were assigned randomly to respondents.

Initially, 1,050 questionnaires were obtained. The questionnaires were examined for data selection. Questionnaires with the following issues were excluded: (a) respondents who have never experienced bike-sharing; (b) the key information was incomplete (*e*.*g*., trip distance or trip purpose); (c) respondents who gave the answer “I don’t know” to satisfaction about bike-sharing questions; (d) cases of contradiction (*e*.*g*., respondent who was young but retired); and (e) cases causing logical problems in the encoding process. A total of, 986 samples were obtained after data selection. The data were analysed anonymously, therefore, the authors had no access to personal identifying information.

Table 1 summarises the cross-tabulation of the respondents’ bike-sharing usage and satisfaction degree of bike-sharing. The bike-sharing usage and satisfaction degree of bike-sharing were defined as a typical ordinal variable that was scaled into three levels (scores): 1—Occasionally (1 to 2 days per week), 2—Generally (3 to 4 days per week), and 3—Frequently (5 to 7 days per week) for bike-sharing usage; 1—Bad, 2—General, and 3—Good for satisfaction degree of bike-sharing. Numerous explanatory variables were collected from the questionnaire to determine whether they influenced bike-sharing usage and level of satisfaction as shown in Tables 2 to 4. The variables of household characteristics, travel patterns, built environments, and perception of bike-sharing are innovative in this study, which are specifically designed for the bike-sharing usage and satisfaction degree of bike-sharing.

**Table 1:** Cross-tabulation by bike-sharing usage and level of satisfaction.

Usage	Satisfaction			Total
	Good	General	Bad	
Frequently	150	73	2	225
Generally	206	171	3	380
Occasionally	158	198	25	381
Total	514	442	30	986

**Table 2:** Summary statistics for individual, and household, characteristic variables.

Variable	Descriptive	Symbol	Frequency	Percentage (%)
***Individual characteristics***				
Gender	Male	1	609	61.76
	Female	0	377	38.24
Age group	Young (12–29)	2	478	48.48
	Middle-aged (30–49)	1	369	37.42
	Older (50–70)	0	139	14.10
Education level	Junior middle school lower	3	42	4.26
	High school and junior middle school	2	276	27.99
	Junior college or undergraduate	1	659	66.84
	Postgraduate and higher	0	9	0.91
Occupation	Student	6	373	37.83
	Employee in enterprise/company	5	314	31.85
	Officer	4	87	8.82
	Self-employed	3	109	11.05
	Freelance	2	53	5.38
	Retired	1	19	1.93
	Others	0	31	3.14
Monthly income	> 5000 (RMB)	3	153	15.52
	3000–5000 (RMB)	2	267	27.08
	2000–3000 (RMB)	1	199	20.10
	< 2000 (RMB)	0	367	37.22
***Household characteristics***				
Car in household	Yes	1	555	56.29
	No	0	431	43.71
Bicycle/e-bike in household	Yes	1	726	73.63
	No	0	260	26.37

**Table 3:** Summary statistics for travel patterns, and built environments variables.

Variable	Descriptive	Symbol	Frequency	Percentage (%)
***Travel patterns***				
Trip mode	Walking	5	234	23.73
	Bicycle	4	148	15.01
	E-bike or motorcycle	3	118	11.97
	Public bus or rail transit	2	244	24.75
	Public transport and bicycle	1	121	12.27
	Car	0	121	12.27
Travel time	< 30 min	2	455	46.15
	30–60 min	1	363	36.82
	> 60 min	0	168	17.04
Trip purpose at weekdays	Go to work	5	566	57.40
	Go to school	4	243	24.65
	Go shopping	3	113	11.46
	See a doctor	2	9	0.91
	Entertainment	1	41	4.16
	Other	0	14	1.42
Trip purpose at weekends	Visiting friends	5	173	17.55
	Go shopping	4	420	42.6
	Travelling	3	102	10.34
	Entertainment	2	176	17.85
	Taking exercise	1	71	7.20
	Other	0	44	4.46
***Built environments***				
Bike-sharing station close to home or workplace*	Yes	1	793	80.43
	No	0	193	19.57
Bus stop close to home or workplace**	Yes	1	902	91.48
	No	0	84	8.52

* The distance from bike-sharing station to home or workplace are within 500 m;

** The distance from bus stop to home or workplace are within 500 m.

**Table 4:** Summary statistics for perception of bike-sharing variables.

Variable	Descriptive	Symbol	Frequency	Percentage (%)
***Perception of bike-sharing***				
Familiarity with bike-sharing*	Yes	1	789	80.00
	No	0	197	20.00
Satisfaction with bike-sharing fee	Yes	1	524	53.14
	No	0	462	46.86
Encouragement of green travel**	Yes	1	767	77.79
	No	0	219	22.21
Saving travel cost by bike-sharing	Yes	1	894	90.67
	No	0	92	9.33
Wasting travel time by bike-sharing	Yes	1	266	26.98
	No	0	720	73.02
Flexible route by bike-sharing***	Yes	1	783	79.41
	No	0	203	20.59
Great effort on the introduction to the public^#^	Yes	1	613	62.17
	No	0	373	37.83
Convenient for applying bike-sharing card	Yes	1	604	61.26
	No	0	382	38.74
Easy to check-in^##^	Yes	1	747	75.76
	No	0	239	24.24
Easy to check-out^##^	Yes	1	698	70.79
	No	0	288	29.21

*Riders know the related policy of bike-sharing, such as the rental costs, how to check in, et al.;

**Riders encourage to use public transit, bikes, walking, and e-bikes when travelling;

***The bike-sharing can provide the riders a flexible route;

^#^ The bike-sharing are greatly introduced to the public, such as the advantages, the policy, and the benefits;

^##^ Check-in/check-out within 5 minutes.

The reliability and validity of the questionnaire was measured using the following methods: Cronbach *α* was used to measure the reliability of the questionnaire [36], the result showed *α* = 0.675 for the overall questionnaire, indicating that the questionnaire could be a sufficiently reliable tool for measuring bike-sharing usage and satisfaction degree of bike-sharing. Face validity and content validity were measured using the expert assessment method. Two experts who have developed many traffic questionnaire surveys were invited to evaluate the readability, feasibility, clarity of wording, layout, and style. A five-point Likert scale was used to measure each item from 1 (bad) to 5 (excellent). The average score for the questionnaire was 4.6, indicating a high face validity of the questionnaire. Average congruency percentage (ACP) was used to measure the context validity of the questionnaire [37]. Experts suggest whether each question on a scale is relevant to the construct, computing the percentage of questions deemed to be relevant for each expert, and then taking an average of the percentages across experts. The average ACP value was 92.3%, indicating a high level of the context validity of the questionnaire.

### Statistical methods

#### Bivariate ordered probit model

The aim of this study is to explore the factors that affect bike-sharing usage and satisfaction degree of bike-sharing. Discrete outcome modelling techniques were utilized as the dependent variables consist of category variables. In particular, the commonly shared unobserved factors that affect both the bike-sharing usage and satisfaction degree of bike-sharing should be accounted for. A BOP model was used to identify factors that affect usage and satisfaction degree of bike-sharing simultaneously. The BOP model is designed to model category dependent variables that may be simultaneously determined [38]. The BOP model starts by defining observed ordinal data for each observation, as given by:

$$\{\begin{array}{lll} y_{i,1}^{*}=\beta_{1}X_{i,1}+\varepsilon_{i,1}, & y_{i,1}=j\;if\;\mu_{j-1}<y_{i,1}^{*}<\mu_{j}, & \;j=0,\cdots J_{1}\\ y_{i,2}^{*}=\beta_{2}X_{i,2}+\varepsilon_{i,2}, & y_{i,2}=k\;if\;\theta_{k-1}<y_{i,2}^{*}<\theta_{k}, & \;k=0,\cdots K_{2} \end{array}$$ (1)

where $y_{i,1}^{*}$ and $y_{i,2}^{*}$ represent latent dependent variables; *y*_*i*,1_ and *y*_*i*,2_ denote the observed outcomes, namely, bike-sharing usage ordinal data (1, 2, 3) and satisfaction degree ordinal data (1, 2, 3); ***X***_*i*,1_ and ***X***_*i*,2_ are vectors containing explanatory variables in the two models; ***β***_1_ and ***β***_2_ are vectors of coefficients associated with explanatory variables in the two models; *μ* and *θ* represent estimated threshold parameters that define *y*_*i*,1_ and *y*_*i*,2_; *ε*_*i*,1_ and *ε*_*i*,2_ represent random error terms for both models, and are normally distributed with mean 0, and variance 1; *ρ* is a correlation coefficient; *i* denotes the observation, and *j* and *k* represent the bike-sharing usage and the satisfaction degree of bike-sharing.

The cross-equation correlated error terms in the BOP model is given by:

$$\left[\begin{array}{c} \varepsilon_{i,1}\\ \varepsilon_{i,2} \end{array}\right]∼N\left(\left[\begin{array}{c} 0\\ 0 \end{array}\right],\left[\begin{array}{cc} 1 & \rho \\ \rho & 1 \end{array}\right]\right)$$ (2)

where *ρ* represents the correlation coefficient between *ε*_*i*,1_ and *ε*_*i*,2_.

Under the assumption of a bivariate normal distribution of random error terms, the joint probability for *y*_i,1_ = *j* and *y*_*i*,2_ = *k* can be expressed as follows:

$$\begin{array}{lll} P(y_{i,1}=j,y_{i,2}=k|X_{i,1},X_{i,2}) & = & \text{Pr}(\mu_{j-1}<y_{i,1}^{*}<\mu_{j};\theta_{k-1}<y_{i,2}^{*}<\theta_{k})\\ & = & \text{Pr}(\mu_{j-1}<\beta_{1}X_{i,1}+\varepsilon_{i,1}<\mu_{j};\theta_{k-1}<\beta_{2}X_{i,2}+\varepsilon_{i,2}<\theta_{k})\\ & = & \text{Pr}(\mu_{j-1}-\beta_{1}X_{i,1}<\varepsilon_{i,1}<\mu_{j}-\beta_{1}X_{i,1};\theta_{k-1}-\beta_{2}X_{i,2}<\varepsilon_{i,2}<\theta_{k}-\beta_{2}X_{i,2})\\ & = & \Phi_{2}[(\mu_{j}-\beta_{1}X_{i,1}),(\theta_{k}-\beta_{2}X_{i,2}),\rho ]-\Phi_{2}[(\mu_{j-1}-\beta_{1}X_{i,1}),(\theta_{k}-\beta_{2}X_{i,2}),\rho ]\\ & & -\Phi_{2}[(\mu_{j}-\beta_{1}X_{i,1}),(\theta_{k-1}-\beta_{2}X_{i,2}),\rho ]+\Phi_{2}[(\mu_{j-1}-\beta_{1}X_{i,1}),(\theta_{k-1}-\beta_{2}X_{i,2}),\rho ] \end{array}$$ (3)

where Φ_2_(.) represents standard bivariate normal cumulative distribution function.

#### BOP model estimation

The parameters estimated in the BOP model are the *J*+*K*-2 threshold values, the coefficients vector ***β***_1_ and ***β***_2_, and the correlation coefficient *ρ*. The parameters can be estimated by maximising the log-likelihood function given by:

$$LL=\sum_{i=1}^{n}(\sum_{j=0}^{J}\sum_{k=0}^{K}\delta_{jk}\left[\begin{array}{c} \Phi_{2}[(\mu_{j}-\beta_{1}X_{i,1}),(\theta_{k}-\beta_{2}X_{i,2}),\rho ]-\Phi_{2}[(\mu_{j-1}-\beta_{1}X_{i,1}),(\theta_{k}-\beta_{2}X_{i,2}),\rho ]\\ -\Phi_{2}[(\mu_{j}-\beta_{1}X_{i,1}),(\theta_{k-1}-\beta_{2}X_{i,2}),\rho ]+\Phi_{2}[(\mu_{j-1}-\beta_{1}X_{i,1}),(\theta_{k-1}-\beta_{2}X_{i,2}),\rho ] \end{array}\right]$$ (4)

where *i* = 1, 2,…, *n* (sample size); *δ*_*jk*_ is defined as being equal to 1 if the observed outcome *y*_*i*_,_1_ = *j* and *y*_*i*_,_2_ = *k*, and zero otherwise.

#### Marginal effects

After model estimation, the signs of the coefficients associated with explanatory variables are of particular interest. The signs indicate the positive or negative effects of the variable on the outcomes. However, the coefficients do not quantify the impacts of these variables, and cannot be intuitively interpreted, especially for intermediate categories. To quantify the effect of each category of outcome, the marginal effects are calculated for variables of interest in the BOP model.

The marginal effect of explanatory variable *X*_*i*,1_ for *y*_*i*,1_ is:

$$\frac{P(y_{i,1}=j)}{\partial X_{i,1}}=\left[\phi (\mu_{j-1}-\beta_{1}X_{i,1})-\phi (\mu_{j}-\beta_{1}X_{i,1})\right]\beta_{1}$$ (5)

where *ϕ*(.) is probability mass function of the standard normal distribution. Similarly, the marginal effect of explanatory variable *X*_*i*,2_ for *y*_*i*,2_ is:

$$\frac{P(y_{i,2}=k)}{\partial X_{i,2}}=\left[\phi (\theta_{k-1}-\beta_{2}X_{i,2})-\phi (\theta_{k}-\beta_{2}X_{i,2})\right]\beta_{2}$$ (6)

## Results and discussion

### Survey results

**Fig 7:**
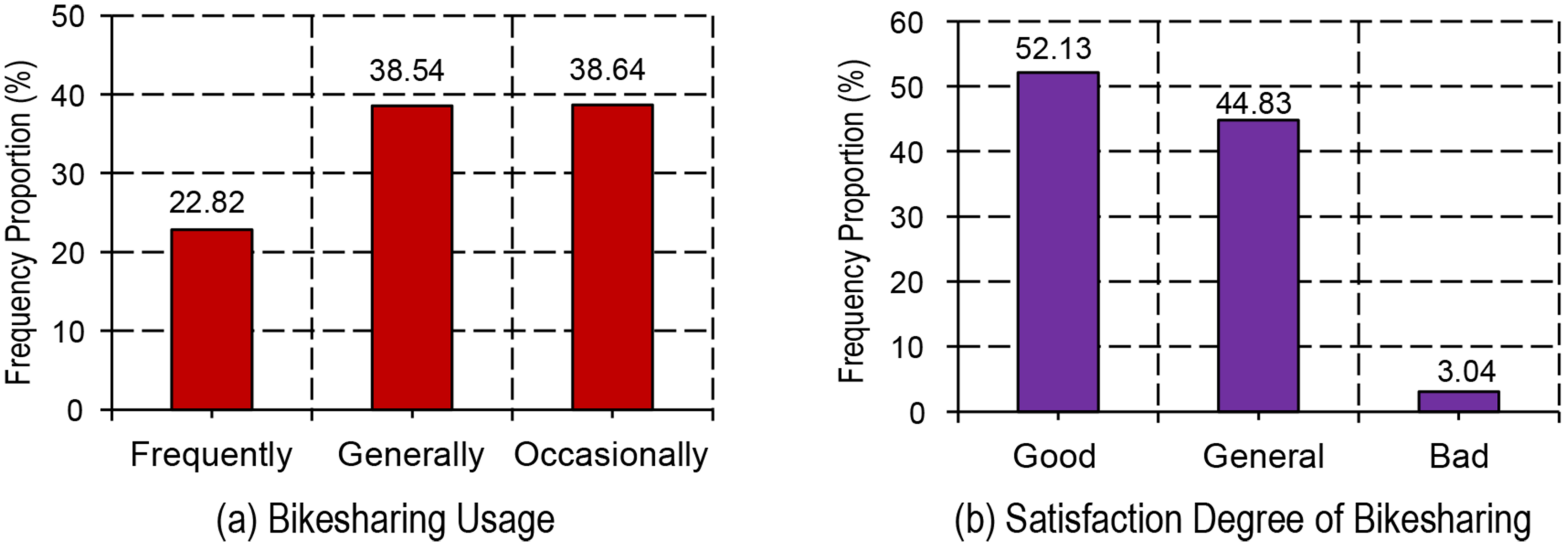
Proportional distribution of usage and satisfaction degree of bike-sharing.

Fig 7 illustrates the proportional distribution of bike-sharing usage and satisfaction degree of bike-sharing across groups. As suggested by Fig 7(a), the respondents who are high-frequency users of the bike-sharing account for 22.8% of the total; while those who usually use the bike-sharing account for 38.5%, which was comparable with those who use bike-sharing occasionally. The results confirmed the low usage of this new mode.

As for the satisfaction degree of bike-sharing, most of the respondents are found to be satisfied with the bike-sharing with a high level (52.1%) (Fig 7(b)). In addition, a slightly lower proportion of respondents seemed to be generally satisfied with the bike-sharing (44.8%). Meanwhile, respondents who tended to be less satisfied account for only a 3.1% of the total. Based on the results, it may be concluded that most of the membership felt a high level of satisfaction with the bike-sharing. Nonetheless, some users who did not satisfy with the bike-sharing should be concerned.

### Model estimation

To identify the factors related to bike-sharing usage and satisfaction degree of bike-sharing, a BOP model was estimated. The explanatory variables and descriptive statistics are shown in Tables 2 to 4. The BOP model estimated result is shown in Table 5. The BOP model showed significant correlation between bike-sharing usage and satisfaction degree of bike-sharing. Only the variables that are significant at a 95% confidence level were included in the final estimated model. The correlation parameter (*ρ* = 0.104, *p*-value = 0.032) was positive, indicating that a higher degree of satisfaction of the bike-sharing can increase the likelihood of bike-sharing usage.

**Table 5:** Estimated results of the BOP model.

Variables	Usage			Satisfaction		
	*β*	S.E.	*p-*value	*β*	S.E.	*p-*value
Gender	0.287	0.083	0.001	—	—	—
Monthly income (2000–3000 RMB)	—	—	—	0.344	0.176	0.049
Monthly income (3000–5000 RMB)	—	—	—	0.596	0.182	0.001
Bicycle/e-bike in household	0.200	0.090	0.027	—	—	—
Trip mode (Bicycle)	0.646	0.156	0.000	—	—	—
Trip mode (Public transport and bicycle)	0.641	0.162	0.000	—	—	—
Travel time (< 30 min)	0.304	0.115	0.008	—	—	—
Bike-sharing station close to home/workplace	0.517	0.115	0.000	0.248	0.114	0.029
Familiarity with bike-sharing	0.636	0.108	0.000	0.542	0.108	0.000
Satisfaction with bike-sharing fees	0.193	0.083	0.019	0.471	0.089	0.000
Encouragement of green travel	0.457	0.100	0.000	—	—	—
Saving travel cost by bike-sharing	—	—	—	0.354	0.142	0.013
Wasting travel time by bike-sharing	-0.243	0.090	0.007	—	—	—
Flexible route by bike-sharing	0.256	0.098	0.009	0.349	0.102	0.001
Great effort on the introduction to the public	0.330	0.087	0.000	0.374	0.094	0.000
Easy to check-in	—	—	—	0.360	0.110	0.001
Easy to check-out	—	—	—	0.242	0.106	0.022
*ρ*	0.104	0.048	0.032			
*μ*_1_	2.655					
*μ*_2_	3.941					
*θ*_1_	-0.874					
*θ*_2_	1.426					
Number of observations	986					
Log likelihood at convergence	-1538.90					

### Model results and discussion

As shown in Table 5, there are twelve significant variables in the bike-sharing usage model and ten variables in the satisfaction degree of bike-sharing model. While the parameters in Table 5 provide a general sense of the direction of impacts of contributing factors on the outcomes, Table 6 shows marginal effects for these variables to quantify their impacts. Since the quantitative impact of each factor on the outcomes can be found from Table 6, the following analysis focused on the outcome of “frequent” bike-sharing usage and a “good” satisfaction degree of bike-sharing.

**Table 6:** Marginal effects for the BOP model.

Bike-sharing usage	Occasionally	Generally	Frequently
Gender	-0.1083	0.0384	0.0699
Bicycle/e-bike in household	-0.0761	0.0279	0.0482
Trip mode (Bicycle)	-0.2176	0.0215	0.1961
Trip mode (Public transport and bicycle)	-0.2139	0.0173	0.1965
Travel time (< 30 min)	-0.1136	0.0359	0.0777
Bike-sharing station close to home/workplace	-0.2012	0.0894	0.1118
Familiarity with bike-sharing	-0.2485	0.1153	0.1332
Satisfaction with bike-sharing fees	-0.0727	0.0242	0.0485
Encouragement of green travel	-0.1779	0.0755	0.1024
Wasting travel time by bike-sharing	0.0937	-0.0350	-0.0586
Flexible route by bike-sharing	-0.0964	0.0374	0.0590
Great effort on the introduction to the public	-0.1255	0.0451	0.0804
Satisfaction Degree of Bike-sharing	Bad	General	Good
Monthly income (2000–3000 RMB)	-0.0069	-0.1274	0.1343
Monthly income (3000–5000 RMB)	-0.0117	-0.2187	0.2304
Bike-sharing station close to home/workplace	-0.0075	-0.0907	0.0982
Familiarity with bike-sharing	-0.0207	-0.1914	0.2120
Satisfaction with bike-sharing fees	-0.0129	-0.1736	0.1865
Saving travel cost by bike-sharing	-0.0126	-0.1270	0.1397
Flexible route by bike-sharing	-0.0113	-0.1263	0.1376
Great effort on the introduction to the public	-0.0127	-0.1573	0.1700
Easy to check-in^△^	-0.0115	-0.1309	0.1423
Easy to check-out^△^	-0.0070	-0.0897	0.0967

Males are generally 7% more likely to use the bike-sharing programme frequently compared to females. The result is consistent with previous work [39] which reported that males were more likely than females to use bike-sharing on trips to work and to access metrorail services. Since bike-sharing was introduced to Ningbo in 2013, her citizens were unfamiliar with this new mode of travel. The result suggests that the bike-sharing programme in Ningbo is preferred by a male-dominated group in its early stages. Respondents who have bicycle/e-bike in household tend to use bike-sharing more frequently. Marginal effects show that the probability of bike-sharing usage for this group is 5% higher than those without bicycle/e-bike. This significant finding suggests that those who have bicycles are more likely to use bike-sharing. The reason may be that bicycle/e-bike owners are more likely to transfer to public bikes to limit financial loss from bike theft and vandalism [14].

Respondents who travelled by bicycle or public transport and bicycle tend to sustain frequent bike-sharing usage. This finding is intuitive because this group who are more accustomed to cycling had high exposure to bike-sharing. Marginal effects show that the probability of bike-sharing usage increases by approximately 20% for people travelling with these two trip modes, indicating that they are potential bike-sharing groups who prefer to switch to this new mode. Furthermore, the results suggest that bike-sharing supports multimodal transport connections [13]. People with a travel time of less than 30 min are 7.77% more likely to use bike-sharing frequently compared to those facing a longer travel time (more than 60 min). The result is consistent with past studies which reported that the travel time by bicycle is generally around 30 min [40,41].

When there is a bike-sharing station available within 500 m of either home or workplace, the probability of bike-sharing usage is found to increase by 11.18%, and the probability of good satisfaction of bike-sharing increases by 9.82%. The result is supported by the study by Bachand-Marleau *et al*. [27] and Austwick *et al*. [42] which reported that bike-sharing system usage increased when there were more bicycle facilities. This finding indicates that easy access (*i*.*e*., a short distance) to a bike-sharing station not only increases the likelihood of bike-sharing usage but also improves the satisfaction degree of bike-sharing. The result offers vital support for bike-sharing system planning and design, especially with regard to bike-sharing station location planning.

People who are familiar with bike-sharing are found to be 13.32% more likely to adopt bike-sharing frequently. The probability of good satisfaction of bike-sharing for this group is found to increase by 21.2%. This finding is straightforward because a better understanding of the advantages and instructions of bike-sharing programmes provide the people more possibility of adopting this mode of transport. People who are satisfied with bike-sharing fees are found to have 4.85% higher likelihood of bike-sharing usage and an 18.65% greater likelihood of good satisfaction of bike-sharing. This finding is consistent with Fishman *et al*. [13] who reported the benefits of bike-sharing to individual financial savings.

Respondents who encourages green travel are 10.24% more likely to use bike-sharing frequently, which may be reflective of their environmental awareness. People who recognizes the flexible route offered them by bike-sharing tend to use it more frequently and are more satisfied with bike-sharing. Marginal effects show that the probability of bike-sharing usage and good satisfaction increased by 5.9% and 13.76%, respectively. However, those who consider reducing their wasted travel time by bike-sharing are 5.86% less likely to adopt it as a preferred mode of transport. A great effort on the introduction of bike-sharing to the public also increases the usage and satisfaction degree of bike-sharing by 8.04% and 17% respectively, indicating an extensive publicity campaign provides sustainable development of the bike-sharing programme.

Some other variables are also found to be associated with satisfaction degree of bike-sharing. Easy to check-in and check-out increases the probability of good satisfaction of bike-sharing by 14.23% and 9.67%, respectively, suggesting that a well-equipped bike-sharing system could increase user satisfaction degree of the system [29]. With the rapid development tread of bike-sharing in China, new sharing bicycles based on mobile intelligent terminals have become very popular. Currently, it is much easier for riders to check-in and check-out at anywhere. The incorporation of mobile intelligent terminals into bike-sharing systems may significantly improve the bike-sharing usage and degree of satisfaction. People who consider bike-sharing as being able to reduce their travel cost are 13.97% more satisfied to the system. The result is consistent with previous findings by Li *et al*. [43] who reported that people trying to save on travel costs prefered cycling. It is interesting to find that monthly income was positively related to satisfaction degree of bike-sharing, which demonstrates that the high income group are 13.43% and 23.04% more satisfied with bike-sharing compared to those with lower incomes. Moreover, according to the coefficient of these two variables, the trend governing changes in level of satisfaction increases with increasing monthly income.

### Improvements for bike-sharing usage

As bike-sharing programmes are booming in Chinese cities, it is important to develop policies or strategies to promote bike-sharing usage. The findings of this study could provide some useful information for proposing improvements to Ningbo’s bike-sharing programme. The findings suggested that an easily available (*i*.*e*., short distance from home or office) bike-sharing station could improve usage; however, in practice, most bike-sharing stations are located at/near shopping centres, metro stations, and bus loops. Since the minor roads connect residential districts to the major roads, more bike-sharing stations could be installed alongside minor roads to improve the availability of bike-sharing.

The results in this study showed that there was a significant positive correlation between bike-sharing usage and satisfaction degree of bike-sharing. As such, a well-designed and well-equipped bike-sharing system is to be encouraged. In the future, the bike-sharing system in Ningbo needs to be upgraded to a fourth-generation service by adopting more smart technology and integrating its operations with other public transport modes.

Some citizens in Ningbo were not familiar with the new system, which hindered its usage. As such, public advocacy for the bike-sharing programme could promote its use. Furthermore, other improvements could be adopted to improve the competitiveness of bike-sharing, such as building favourable, more enjoyable, cycling environments, designing more continuous bicycle lanes or paths, and increasing the number of bike-sharing stations.

## Conclusions

Although bike-sharing is widely used as a commuter traffic mode in China, little is known regarding the factors related to bike-sharing usage and satisfaction degree of bike-sharing. This present study applied a BOP model to identify those factors affecting bike-sharing usage and user satisfaction degree of bike-sharing. Data were collected, in Ningbo, using a questionnaire survey approach. The survey results showed that most of the memberships were highly satisfied with the bike-sharing. However, the usage of bike-sharing remained relatively low. A total of 986 valid samples were used to develop the BOP model. Furthermore, this study provided several improvement measures for bike-sharing usage based on the modelling results.

The estimated model results showed several contributory variables. In the bike-sharing usage model, twelve variables were found to be statistically significant, which were ranked by importance: trip mode (bicycle, public transport and bicycle), familiarity with bike-sharing, bike-sharing station location, encouragement of green travel, great effort on the introduction to the public, travel time (< 30 min), gender, flexible route by bike-sharing, wasting travel time by bike-sharing, household bicycle/e-bike ownership, and satisfaction with bike-sharing fees. In the satisfaction model, ten variables were found to be significant, which were ranked by importance: monthly income (3000–5000 RMB, 2000–3000 RMB), familiarity with bike-sharing, satisfaction with bike-sharing fees, great effort on the introduction to the public, easy to check-in and check-out, saving travel cost by bike-sharing, flexible route by bike-sharing, and bike-sharing station location.

The findings of this study provided insight into the factors associated with usage and satisfaction degree of bike-sharing. In China, many governments in large cities have invested heavily to launch their bike-sharing programmes. However, the usage of bike-sharing in some cities was unsatisfactory. The low usage of bike-sharing is a key problem hindering sustainable development of this traffic mode. A better understanding of the factors affecting usage and satisfaction degree of bike-sharing could provide solutions to this problem. Based on the findings in this study, several useful improvements for promoting bike-sharing usage were discussed. The policies could also help persuade travellers to become bike-sharing commuters.

The BOP model allowed for consideration of cross-equation correlation between bike-sharing usage and satisfaction of bike-sharing. The estimated results showed that the correlation parameter was statistically significant, indicating that several commonly shared unobserved characteristics were captured by the error terms of the two latent variables. In addition, the positive correlation parameter implied that a higher degree of satisfaction of bike-sharing can increase the likelihood of bike-sharing usage. Furthermore, marginal effects for contributory variables were calculated to quantify their impacts on the outcomes.

There are some limitations to the present study. The survey was only conducted in Ningbo city, however, the operations of bike-sharing programmes varied across other cities. In some cities such as Hangzhou, the bike-sharing project is very successful, but, in some other cities, such as Nanjing, bike-sharing usage is quite low. Studies based on multiple cities could help better understand and capture more factors affecting usage and satisfaction degree of bike-sharing. Additionally, bike-sharing based on mobile intelligent terminals have become very popular in China, an update questionnaire survey could be conducted to capture more meaningful factor with bike-sharing usage. Furthermore, the extension of this work should examine the unobserved heterogeneity across bike-sharing users. Recent work provided the framework of a random parameter BOP model [44]. Under this framework, parameter effects on usage and satisfaction degree of bike-sharing across users can be estimated.

## Supporting information

S1 Data Data used for figures. (XLSX)
